# Could R‐Ketamine and Wolfram Syndrome Inform Understanding of Depression and Suicidality? A Sigma‐1 Receptor‐Based Perspective

**DOI:** 10.1002/hup.70019

**Published:** 2025-09-16

**Authors:** Hans O. Kalkman, Lukasz Smigielski

**Affiliations:** ^1^ Department of Child and Adolescent Psychiatry and Psychotherapy University Hospital of Psychiatry University of Zurich Zurich Switzerland

**Keywords:** σ1 agonist, ketamine enantiomer, mitochondria‐associated ER membranes, neuronal Ca^2+^sensor‐1, suicide, wolframin

## Abstract

Loss of function mutations in the *WFS1* gene cause Wolfram syndrome, which is characterized by juvenile‐onset diabetes mellitus, diabetes insipidus, neurodegeneration, hearing loss and optic nerve atrophy. Psychiatric symptoms, including major depression and suicidal behavior, are common in this disorder. *WFS1* mutations induce this condition through altering interactions between the endoplasmic reticulum and mitochondria, resulting in diminished Ca^2+^ import that leads to mitochondrial dysfunction. Quite recently, it was shown that such impaired Ca^2+^ transport could be restored by the experimental σ1 receptor agonist PRE084. In animal models of Wolfram syndrome, this compound restored the behavioral phenotype. Based on these previous data, we propose that Wolfram syndrome may serve as a mechanistically informative model for exploring σ1 receptor modulation, mitochondrial dysfunction, and affective symptoms. This proposal is based on four arguments. Firstly, the R‐enantiomer of ketamine exhibits largely selective binding to the σ1 receptor as an agonist. Secondly, R‐ketamine and other σ1 agonists display antidepressant‐like activity in rodent depression models. Thirdly, while both S‐ and R‐ketamine hold potential for reducing suicidal behavior, the latter is likely to have a lower potential for abuse and fewer side effects. Fourth, Wolfram syndrome is characterized by mitochondrial dysfunction, which has also been linked to depression.

## 1 Background

### 1.1 Wolfram Syndrome

Wolfram syndrome is a rare autosomal recessive disorder affecting the brain and multiple other organs (Caruso et al. 2024; Kõks 2023). Its pathology is characterized by juvenile‐onset diabetes mellitus, diabetes insipidus, neurodegeneration, hearing loss and optic nerve atrophy (Chaussenot et al. 2011; R. G. Swift et al. 1990). Additionally, individuals who are affected by Wolfram syndrome often present an increased risk of psychiatric disorders, particularly depression and suicidal behavior (Cryns et al. 2003; Koido et al. 2005; Mirfazeli et al. 2022; Sequeira et al. 2003; R. G. Swift et al. 1990). Carriers of the *WS* gene were found to be 26 times more likely to require psychiatric hospitalization than non‐carriers, and the incidence of suicide‐related behaviors in Wolfram syndrome was found to be high, reaching 25% (R. G. Swift et al. 1998). In addition, the relative risk of psychiatric hospitalization due to depression was estimated at 7.1 (M. Swift and Swift 2005). Furthermore, the disease causes significant early‐stage brain abnormalities (Hershey et al. 2012) and later neurodegenerative effects (Rigoli et al. 2018) involving mechanisms known to contribute to the development of psychiatric disorders (Cagalinec et al. 2016; Hao et al. 2023). Overall, this suggests that while affective symptoms in Wolfram syndrome may in part reflect the burden of medical illness, they could also be linked to mechanisms that are driven by the syndrome's core pathophysiological features.

Wolfram syndrome is caused by mutations in *WFS1*, a gene that encodes the glycoprotein wolframin (Crouzier et al. 2022). Wolframin is localized to particular areas of the endoplasmic reticulum (ER) that interact with mitochondria, which are referred to as mitochondria‐associated ER membranes (MAMs) (Crouzier et al. 2022). Within MAMs, wolframin interacts with two MAM‐resident proteins: neuronal Ca^2+^ sensor‐1 (NCS1) and inositol‐triphosphate‐receptor‐1 (IP3R1) (Angebault et al. 2018). Wolframin is furthermore known to interact with voltage‐dependent anion channel‐1 (VDAC1), a protein that is located in the outer leaflet of the mitochondrial membrane (Zatyka et al. 2023). Mutations in WFS1 affect protein stability (Kõks 2023) and diminish IP3R‐VDAC1 dependent Ca^2+^ trafficking between the ER and the mitochondrial matrix (Angebault et al. 2018; Delprat et al. 2018; Liiv et al. 2024). This has severe functional consequences, including mitochondrial dysfunction, reduced ATP production, increased production of reactive oxygen radicals and increased apoptotic cell death (Crouzier et al. 2022; Zatyka et al. 2023). Mitochondrial dysfunction and its pro‐apoptotic consequences provide a plausible explanation for general neurodegeneration, the loss of optic, acoustic and hypothalamic vasopressin neurons, as well as the destruction of pancreatic *β*‐cells. Reactive oxygen species are known to activate c‐JUN N‐terminal kinase (JNK), which inhibits the insulin‐signal transduction, thereby contributing to insulin resistance and the development of type 2 diabetes (Aguirre et al. 2002; Yin et al. 2013). Furthermore, major depression is associated—and possibly caused by—mitochondrial dysfunction (Allen et al. 2021; Bansal and Kuhad 2016; Cardon et al. 2024). The pathophysiological process underlying suicide, however, remains largely unknown. This is partly due to the inability to model suicide in animal studies, and the low incidence of fatal suicide (even in psychiatric populations). As a nearly monogenetic disorder (Caruso et al. 2024), Wolfram syndrome may offer unique insight in the pathophysiology of suicide and, potentially, severe depression. Given the urgent need for a deeper understanding of suicide, the notably high incidence of suicide in Wolfram syndrome may provide insights into the biological mechanisms underlying suicidality. There is currently no specific treatment for the metabolic and CNS pathologies associated with WFS1 mutations (Caruso et al. 2024; Kõks 2023).

### 1.2 Wolframin Interacts With NCS1 and σ1 Receptors

Quite recently, Crouzier and colleagues reported that the experimental compound PRE084, a selective agonist of σ1 receptors (Motawe et al. 2020), restored mitochondrial respiration and alleviated behavioral symptoms in animal models of Wolfram syndrome (Crouzier et al. 2022). The σ1 receptor is a small, ligand‐regulated chaperone protein (Ossa et al. 2017; Szabo et al. 2016) that modulates cellular Ca^2+^ homeostasis through interactions with several ligand‐ and voltage‐gated ion channels, including IP3R (Hayashi and Su 2007; Schmidt and Kruse 2019). Moreover, σ1 receptors are densely clustered within MAMs (Hayashi and Su 2003), and upon ligand binding, they potentiate Ca^2+^ flux from the ER to mitochondria (Hayashi 2019; Ossa et al. 2017; Szabo et al. 2016). After entry into the mitochondrion, Ca^2+^ drives the Krebs cycle, boosts the mitochondrial respiratory chain and thus raises ATP production (Bonora et al. 2012; Patergnani et al. 2014). Notably, the identity of the endogenous ligand of the σ1 receptor remains unclear (Pergolizzi et al. 2023). Proposed candidates include choline, neuro‐steroids, dimethyltryptamines, myristic acid and sphingolipids (Brailoiu et al. 2019; Fu et al. 2024; Szabo et al. 2016). In the central nervous system, σ1 receptors are expressed by neurons, microglia, astrocytes and oligodendrocytes (Nguyen et al. 2015).

As noted, at ER–mitochondrion interfaces, wolframin forms a complex with NCS1, IP3R and VDAC1 to promote Ca^2+^ transfer between the ER and mitochondria (Angebault et al. 2018; Zatyka et al. 2023). The point of convergence between the proteins that interact with wolframin and with σ1 receptors is NCS1. In Neuro2A cells, σ1 receptors coprecipitated with NCS1 and more faintly also with wolframin (Crouzier et al. 2022). In fibroblasts from patients with Wolfram syndrome, the expression of NCS1 in MAMs was reduced, whereas overexpression of NCS1 or activation of σ1 receptors restored the diminished Ca^2+^ transfer and its functional consequences (Crouzier et al. 2022). Contrary to its name, NCS1 is expressed beyond neurons (and fibroblasts), as it is also expressed by cardiomyocytes, pancreatic *β*‐cells, retinal ganglion cells (summarized in Angebault et al. 2018), neuroendocrine cells, mast cells, neutrophils, lymphocytes and monocytes (Torres et al. 2009), as well as in different cancer cell lines (Sánchez et al. 2024). As a putative indicator of MAM dysfunction, altered expression of NCS1 was reported in tissues from patients with bipolar disorder and schizophrenia (Koh et al. 2003; Torres et al. 2009).

Whilst selective agonists for the σ1 receptor are under clinical development (Malar et al. 2023), only a few have reached the clinical research stage and none have entered the market (Crouzier et al. 2022). Nevertheless, several medications, including donepezil, rivastigmine, fenfluramine, fluoxetine, fluvoxamine, ketamine and memantine, display, in addition to their targeted pharmacological activity, a relevant σ1 agonist effect (Malar et al. 2023; Robson et al. 2012). Such compounds could serve as pharmacological tools for pilot studies in human subjects.

## 2 Ketamine's R‐Enantiomer Displays Significant Affinity for σ1 Receptors

Ketamine is a rapidly acting antidepressant compound with efficacy in treatment‐resistant depression (Alnefeesi et al. 2022; Bahji et al. 2021; Bentley et al. 2022; Fava et al. 2020; Marcantoni et al. 2020). There is also a rich literature indicating that ketamine effectively reduces suicidal behavior (Averill et al. 2022; Ballard et al. 2014; Feeney et al. 2021; Price et al. 2014; Wilkinson et al. 2018). Ketamine is a racemate. S‐ketamine blocks ion transport through the NMDA‐type glutamate‐gated ion channel and acts as a partial agonist activity of opioid *μ*‐receptors (Bonaventura et al. 2021). Alongside the widely studied S‐ketamine in clinical settings, the less‐researched R‐enantiomer of ketamine (also known as arketamine) is emerging as a potential alternative for treatment‐resistant depression (Scotton et al. 2022). In animal studies, R‐ketamine demonstrated higher potency and more prolonged antidepressant effects compared to S‐ketamine (Fukumoto et al. 2017; J. C. Zhang et al. 2014). In humans, limited small studies have observed a rapid antidepressant effect of R‐ketamine infusions in treatment‐resistant depressive patients (Leal et al. 2021), with benefits extending over longer follow‐up periods (Włodarczyk et al. 2024). However, a subsequent small placebo‐controlled study by the same group (*n* = 10) did not demonstrate superiority of R‐ketamine over placebo, and larger confirmatory trials are still lacking (Johnston et al. 2023). Unpublished results from a 2023 Phase 2a trial showed no statistically significant difference in depressive symptoms between R‐ketamine (30 and 60 mg) and placebo at the 24‐h primary endpoint, though numerically greater response and remission rates were observed in the 60 mg arm over 2 weeks (Atai Life Sciences 2023, press release). In light of this still limited human evidence, our discussion of R‐ketamine is framed within a hypothesis‐generating and mechanistic context, not as a therapeutic recommendation.

Additionally, R‐ketamine, in contrast to S‐ketamine, does not profoundly induce psychotomimetic effects (Hashimoto 2020; Vollenweider et al. 1997) or dissociation (Leal et al. 2021) and is likely to have a lower potential for abuse (Chang et al. 2019). Both S‐ and R‐ketamine hold the potential as rapid antisuicidal agents (Dadiomov and Lee 2019). However, distinctions between these two enantiomers may produce different outcomes in terms of their effects on suicidality. Of note, the effects of R‐ketamine are mediated via activation of the extracellular signal‐regulated kinase (ERK) pathway, whereas the effects of S‐ketamine are mediated through disinhibition of mechanistic target of rapamycin complex 1 (mTORC1) signaling (Kang et al. 2022; Yang et al. 2015). Notably, R‐ketamine displays significant affinity for σ1 receptors (Bonaventura et al. 2021; Hustveit et al. 1995), whereas it lacks relevant affinity to other receptor binding sites (Bonaventura et al. 2021). Presumably, R‐ketamine is a σ1 agonist, as in a drug‐discrimination study the ketamine‐induced drug cue was inhibited by a σ1 receptor antagonist (Narita et al. 2001). Moreover, in some preclinical depression models, the antidepressant‐like responses of ketamine were blocked by σ1 receptor antagonists (Ma et al. 2024; Robson et al. 2012).

## 3 σ1 Receptors at the Crossroads of Metabolic and Psychiatric Dysregulation

The signaling process by which mutated WFS1 proteins give rise to diabetes or to depressive and suicidal behaviors remains to be detailed, but most likely involves mitochondrial dysfunction caused by reduced Ca^2+^ transport and altered interactions between the ER and mitochondria in pancreatic *β*‐cells and neurons, respectively (Angebault et al. 2018). Data from Crouzier et al. (2022) provide the critical insight that impaired Ca^2+^ transport across the MAMs can be rescued by PRE084, an agonist of σ1 receptors. Since functional mitochondria are essential for both cellular function and survival, it is unsurprising that the activation of σ1 receptors has been proposed as a potential treatment for behavioral/psychiatric disorders (Ren et al. 2022; Salaciak and Pytka 2022; Voronin et al. 2020; Y. M. Wang et al. 2022) and neurological/neurodegenerative disorders (Herrando‐Grabulosa et al. 2021; Malar et al. 2023; Weng et al. 2017). R‐ketamine seems to be a selective σ1 agonist, and in preclinical studies, R‐ketamine indeed displayed antidepressant‐like activity (Hashimoto 2020; J. Zhang et al. 2021; Johnston et al. 2023). However, clinical confirmation that R‐ketamine possesses antidepressant activity remains lacking; in contrast, the anti‐depressant effect of S‐ketamine has been clearly established (Chen et al. 2023; Correia‐Melo et al. 2020; Singh et al. 2023; Takahashi et al. 2021). Whether stimulation of σ1 receptors reduces insulin resistance has not been investigated in humans or even in animal models. Since σ1 agonists improve mitochondrial function and presumably reduce the production of reactive oxygen radicals (Goguadze et al. 2019; Oxombre et al. 2015), it is possible that σ1 agonists counteract insulin‐resistance by restoring insulin‐induced signal transduction.

In this context, it is worth noting that the antidepressant effect of racemic ketamine was relatively greater both in patients with a high versus low body mass index (BMI) (Freeman et al. 2020) and in those with low versus high adiponectin levels (Machado‐Vieira et al. 2017). Both high BMI values and low adiponectin levels are indicators of type 2 diabetes and thus insulin resistance (Hotta et al. 2001; Kopp et al. 2005; Y. Wang et al. 2018). This observation is consistent with data from an animal study in which the SSRI/σ1 agonist fluvoxamine inhibited depression‐like behavior in streptozotocin‐induced diabetic rats, an effect that was blocked by a σ1 antagonist (Lenart et al. 2016). Patients with depression and suicidal behavior were reported to exhibit higher blood glucose concentrations than depressed patients without suicidal behavior, and glucose levels were found to correlate with the prevalence of suicidal behavior (Koponen et al. 2015). Similarly, patients who had made violent suicide attempt showed higher CSF insulin levels than those with nonviolent attempts (Westling et al. 2004), as well as elevated plasma and CSF insulin levels compared to healthy controls (Bendix et al. 2017). These results point to a role of insulin resistance in suicidal behavior, which may, in some cases, be independent of depression (Westling et al. 2004).

## 4 Future Directions

Based on the aforementioned circumstantial evidence, clinicians could reasonably consider fluvoxamine treatment of patients with Wolfram syndrome and its associated psychiatric symptoms. However, it remains unclear if the target therapeutic effect would be caused by 5‐HT uptake inhibition or σ1 stimulation. Wolfram syndrome also provides a valuable model to investigate the therapeutic potential of repairing mitochondrial dysfunction, possibly mediated by sigma‐1 receptor pathways. While clinical application is premature at this stage, exploratory studies with R‐ketamine in patients with Wolfram syndrome could help to partially elucidate these mechanisms and generate further hypotheses. Given the severity and lethality of Wolfram syndrome (Kinsley et al. 1995), and the absence of effective treatments, such exploratory investigations may be justified and hold translational potential. This line of research also has the potential to elucidate other indications in which σ1 stimulation and downstream pathways may be of therapeutic value. It should be noted that other compounds are also of interest in Wolfram syndrome. Dantrolene, an ER calcium stabilizer, has been shown to reduce ER stress and prevent cell death in WFS1‐deficient iPSC‐derived neural progenitor cells (Lu et al. 2014), and has demonstrated safety and potential benefit in a Phase Ib/IIa clinical trial (Abreu et al. 2021). Valproic acid, a mood stabilizer that induces p21 and WFS1 expression, confers protection against cell death, modulates ER stress, and prevents aberrant axon guidance and neurite outgrowth defects (Gharanei et al. 2013; Kakiuchi et al. 2009; Pourtoy‐Brasselet et al. 2021), and is currently under investigation in a Phase 2 clinical trial (NCT03717909; Dias et al. 2025). GLP‐1 receptor agonists have also been shown to alleviate ER stress, support *β*‐cell survival, and reduce neurodegeneration in rodent and human preclinical models of Wolfram syndrome (Gorgogietas et al. 2023; Kondo et al. 2018; Seppa et al. 2019).

## Conflicts of Interest

The authors declare no conflicts of interest.

## Data Availability

Data sharing is not applicable, as no datasets were generated or analyzed in the preparation of this article.

## References

[hup70019-bib-0001] Abreu, D. , S. I. Stone , T. S. Pearson , et al. 2021. “A Phase Ib/IIa Clinical Trial of Dantrolene Sodium in Patients With Wolfram syndrome.” JCI Insight 6, no. 15: e145188. 10.1172/jci.insight.145188. 34185708 PMC8410026

[hup70019-bib-0002] Aguirre, V. , E. D. Werner , J. Giraud , Y. H. Lee , S. E. Shoelson , and M. F. White . 2002. “Phosphorylation of Ser307 in Insulin Receptor substrate‐1 Blocks Interactions With the Insulin Receptor and Inhibits Insulin Action.” Journal of Biological Chemistry 277, no. 2: 1531–1537. 10.1074/jbc.M101521200. 11606564

[hup70019-bib-0003] Allen, J. , H. J. Caruncho , and L. E. Kalynchuk . 2021. “Severe Life Stress, Mitochondrial Dysfunction, and Depressive Behavior: A Pathophysiological and Therapeutic Perspective.” Mitochondrion 56: 111–117. 10.1016/j.mito.2020.11.010. 33220501

[hup70019-bib-0004] Alnefeesi, Y. , D. Chen‐Li , E. Krane , et al. 2022. “Real‐World Effectiveness of Ketamine in Treatment‐Resistant Depression: A Systematic Review and Meta‐Analysis.” Journal of Psychiatric Research 151: 693–709. 10.1016/j.jpsychires.2022.04.037. 35688035

[hup70019-bib-0005] Angebault, C. , J. Fauconnier , S. Patergnani , et al. 2018. “ER‐Mitochondria Cross‐Talk is Regulated by the Ca2+ Sensor NCS1 and is Impaired in Wolfram syndrome.” Science Signaling 11, no. 553: eaaq1380. 10.1126/scisignal.aaq1380. 30352948

[hup70019-bib-0006] Atai Life Sciences . 2023. Atai Life Sciences Announces Results From Phase 2a Trial of PCN‐101 (R‐Ketamine) for Treatment‐Resistant Depression. GlobeNewswire. https://ir.atai.life/news‐releases/news‐release‐details/atai‐life‐sciences‐announces‐results‐phase‐2a‐trial‐pcn‐101‐r.

[hup70019-bib-0007] Averill, L. A. , C. L. Averill , R. Gueorguieva , et al. 2022. “mTORC1 Inhibitor Effects on Rapid Ketamine‐Induced Reductions in Suicidal Ideation in Patients With Treatment‐Resistant Depression.” Journal of Affective Disorders 303: 91–97. 10.1016/j.jad.2022.01.104. 35101523

[hup70019-bib-0008] Bahji, A. , G. H. Vazquez , and C. A. Zarate . 2021. “Comparative Efficacy of Racemic Ketamine and Esketamine for Depression: A Systematic Review and Meta‐Analysis.” Journal of Affective Disorders 278: 542–555. 10.1016/j.jad.2020.09.071. 33022440 PMC7704936

[hup70019-bib-0009] Ballard, E. D. , D. F. Ionescu , J. L. Vande Voort , et al. 2014. “Improvement in Suicidal Ideation After Ketamine Infusion: Relationship to Reductions in Depression and Anxiety.” Journal of Psychiatric Research 58: 161–166. 10.1016/j.jpsychires.2014.07.027. 25169854 PMC4163501

[hup70019-bib-0010] Bansal, Y. , and A. Kuhad . 2016. “Mitochondrial Dysfunction in Depression.” Current Neuropharmacology 14, no. 6: 610–618. 10.2174/1570159x14666160229114755. 26923778 PMC4981740

[hup70019-bib-0011] Bendix, M. , K. Uvnäs‐Moberg , M. Petersson , V. Kaldo , M. Åsberg , and J. Jokinen . 2017. “Insulin and Glucagon in Plasma and Cerebrospinal Fluid in Suicide Attempters and Healthy Controls.” Psychoneuroendocrinology 81: 1–7. 10.1016/j.psyneuen.2017.03.019. 28391069

[hup70019-bib-0012] Bentley, S. , H. Artin , E. Mehaffey , et al. 2022. “Response to Intravenous Racemic Ketamine After Switch From Intranasal (S)‐Ketamine on Symptoms of treatment‐resistant Depression and Post‐Traumatic Stress Disorder in Veterans: A Retrospective Case Series.” Pharmacotherapy: The Journal of Human Pharmacology and Drug Therapy 42, no. 3: 272–279. 10.1002/phar.2664. PMC8934379 35122282

[hup70019-bib-0013] Bonaventura, J. , S. Lam , M. Carlton , et al. 2021. “Pharmacological and Behavioral Divergence of Ketamine Enantiomers: Implications for Abuse Liability.” Molecular Psychiatry 26, no. 11: 6704–6722. 10.1038/s41380-021-01093-2. 33859356 PMC8517038

[hup70019-bib-0014] Bonora, M. , S. Patergnani , A. Rimessi , et al. 2012. “ATP Synthesis and Storage.” Purinergic Signalling 8, no. 3: 343–357. 10.1007/s11302-012-9305-8. 22528680 PMC3360099

[hup70019-bib-0015] Brailoiu, E. , S. Chakraborty , G. C. Brailoiu , et al. 2019. “Choline is an Intracellular Messenger Linking Extracellular Stimuli to IP3‐evoked Ca2+ Signals Through Sigma‐1 Receptors.” Cell Reports 26, no. 2: 330–337.e4. 10.1016/j.celrep.2018.12.051. 30625315 PMC6326163

[hup70019-bib-0016] Cagalinec, M. , M. Liiv , Z. Hodurova , et al. 2016. “Role of Mitochondrial Dynamics in Neuronal Development: Mechanism for Wolfram syndrome.” PLoS Biology 14, no. 7: e1002511. 10.1371/journal.pbio.1002511. 27434582 PMC4951053

[hup70019-bib-0017] Cardon, I. , S. Grobecker , F. Jenne , et al. 2024. “Serotonin Effects on Human iPSC‐derived Neural Cell Functions: From Mitochondria to Depression.” Molecular Psychiatry 29, no. 9: 2689–2700. 10.1038/s41380-024-02538-0. 38532010 PMC11420088

[hup70019-bib-0018] Caruso, V. , A. Raia , and L. Rigoli . 2024. “Wolfram syndrome 1: A Neuropsychiatric Perspective on a Rare Disease.” Genes 15, no. 8: 984. 10.3390/genes15080984. 39202345 PMC11353439

[hup70019-bib-0019] Chang, L. , K. Zhang , Y. Pu , et al. 2019. “Comparison of Antidepressant and Side Effects in Mice After Intranasal Administration of (R,S)‐Ketamine, (R)‐Ketamine, and (S)‐Ketamine.” Pharmacology Biochemistry and Behavior 181: 53–59. 10.1016/j.pbb.2019.04.008. 31034852

[hup70019-bib-0020] Chaussenot, A. , S. Bannwarth , C. Rouzier , et al. 2011. “Neurologic Features and Genotype‐Phenotype Correlation in Wolfram syndrome.” Annals of Neurology 69, no. 3: 501–508. 10.1002/ana.22160. 21446023

[hup70019-bib-0021] Chen, C. C. , N. Zhou , N. Hu , J. G. Feng , and X. B. Wang . 2023. “Acute Effects of Intravenous Sub‐Anesthetic Doses of Ketamine and Intranasal Inhaled Esketamine on Suicidal Ideation: A Systematic Review and Meta‐Analysis.” Neuropsychiatric Disease and Treatment 19: 587–599. 10.2147/NDT.S401032. 36942150 PMC10024508

[hup70019-bib-0022] Correia‐Melo, F. S. , G. C. Leal , F. Vieira , et al. 2020. “Efficacy and Safety of Adjunctive Therapy Using Esketamine or Racemic Ketamine for Adult Treatment‐Resistant Depression: A Randomized, Double‐Blind, Non‐Inferiority Study.” Journal of Affective Disorders 264: 527–534. 10.1016/j.jad.2019.11.086. 31786030

[hup70019-bib-0023] Crouzier, L. , A. Danese , Y. Yasui , et al. 2022. “Activation of the Sigma‐1 Receptor Chaperone Alleviates Symptoms of Wolfram Syndrome in Preclinical Models.” Science Translational Medicine 14, no. 631: eabh3763. 10.1126/scitranslmed.abh3763. 35138910 PMC9516885

[hup70019-bib-0024] Cryns, K. , T. A. Sivakumaran , J. M. Van den Ouweland , et al. 2003. “Mutational Spectrum of the WFS1 Gene in Wolfram Syndrome, Nonsyndromic Hearing Impairment, Diabetes Mellitus, and Psychiatric Disease.” Human Mutation 22, no. 4: 275–287. 10.1002/humu.10258. 12955714

[hup70019-bib-0025] Dadiomov, D. , and K. Lee . 2019. “The Effects of Ketamine on Suicidality Across Various Formulations and Study Settings.” Mental Health Clinician 9, no. 1: 48–60. 10.9740/mhc.2019.01.048. 30627504 PMC6322816

[hup70019-bib-0026] Delprat, B. , T. Maurice , and C. Delettre . 2018. “Wolfram Syndrome: Mams' Connection?” Cell Death & Disease 9, no. 3: 364. 10.1038/s41419-018-0406-3. 29511163 PMC5840383

[hup70019-bib-0027] Dias, R. P. , K. Brock , K. Hu , et al. 2025. “Sodium Valproate, a Potential Repurposed Treatment for the Neurodegeneration in Wolfram syndrome (TREATWOLFRAM): Trial Protocol for a Pivotal Multicentre, Randomised Double‐Blind Controlled Trial.” BMJ Open 15, no. 2: e091495. 10.1136/bmjopen-2024-091495. PMC11865774 40010822

[hup70019-bib-0028] Fava, M. , M. P. Freeman , M. Flynn , et al. 2020. “Double‐Blind, Placebo‐Controlled, Dose‐Ranging Trial of Intravenous Ketamine as Adjunctive Therapy In Treatment‐Resistant Depression (TRD).” Molecular Psychiatry 25, no. 7: 1592–1603. 10.1038/s41380-018-0256-5. 30283029 PMC6447473

[hup70019-bib-0029] Feeney, A. , R. S. Hock , M. P. Freeman , et al. 2021. “The Effect of Single Administration of Intravenous Ketamine Augmentation on Suicidal Ideation in Treatment‐Resistant Unipolar Depression: Results From a Randomized Double‐Blind Study.” European Neuropsychopharmacology 49: 122–132. 10.1016/j.euroneuro.2021.04.024. 34090255 PMC8338746

[hup70019-bib-0030] Freeman, M. P. , R. S. Hock , G. I. Papakostas , et al. 2020. “Body Mass Index as a Moderator of Treatment Response to Ketamine for Major Depressive Disorder.” Journal of Clinical Psychopharmacology 40, no. 3: 287–292. 10.1097/JCP.0000000000001209. 32332464 PMC7185034

[hup70019-bib-0031] Fu, C. , Y. Xiao , X. Zhou , and Z. Sun . 2024. “Insight into Binding of Endogenous Neurosteroid Ligands to the Sigma‐1 Receptor.” Nature Communications 15, no. 1: 5619. 10.1038/s41467-024-49894-7. PMC11224282 38965213

[hup70019-bib-0032] Fukumoto, K. , H. Toki , M. Iijima , et al. 2017. “Antidepressant Potential of (R)‐Ketamine in Rodent Models: Comparison With (S)‐Ketamine.” Journal of Pharmacology and Experimental Therapeutics 361, no. 1: 9–16. 10.1124/jpet.116.239228. 28115553

[hup70019-bib-0033] Gharanei, S. , M. Zatyka , D. Astuti , et al. 2013. “Vacuolar‐Type H+‐ATPase V1A Subunit is a Molecular Partner of Wolfram syndrome 1 (WFS1) Protein, Which Regulates Its Expression and Stability.” Human Molecular Genetics 22, no. 2: 203–217. 10.1093/hmg/dds400. 23035048

[hup70019-bib-0034] Goguadze, N. , E. Zhuravliova , D. Morin , D. Mikeladze , and T. Maurice . 2019. “Sigma‐1 Receptor Agonists Induce Oxidative Stress in Mitochondria and Enhance Complex I Activity in Physiological Condition but Protect Against Pathological Oxidative Stress.” Neurotoxicity Research 35, no. 1: 1–18. 10.1007/s12640-017-9838-2. 29127580

[hup70019-bib-0035] Gorgogietas, V. , B. Rajaei , C. Heeyoung , et al. 2023. “GLP‐1R Agonists Demonstrate Potential to Treat Wolfram syndrome in Human Preclinical Models.” Diabetologia 66, no. 7: 1306–1321. 10.1007/s00125-023-05905-8. 36995380 PMC10244297

[hup70019-bib-0036] Hao, H. , L. Song , and L. Zhang . 2023. “Wolfram Syndrome 1 Regulates Sleep in Dopamine Receptor Neurons by Modulating Calcium Homeostasis.” PLoS Genetics 19, no. 7: e1010827. 10.1371/journal.pgen.1010827. 37399203 PMC10348591

[hup70019-bib-0037] Hashimoto, K. 2020. “Molecular Mechanisms of the Rapid‐Acting and Long‐Lasting Antidepressant Actions of (R)‐ketamine.” Biochemical Pharmacology 177: 113935. 10.1016/j.bcp.2020.113935. 32224141

[hup70019-bib-0038] Hayashi, T. 2019. “The sigma‐1 Receptor in Cellular Stress Signaling.” Frontiers in Neuroscience 13: 733. 10.3389/fnins.2019.00733. 31379486 PMC6646578

[hup70019-bib-0039] Hayashi, T. , and T. P. Su . 2003. “Sigma‐1 Receptors (Sigma(1) Binding Sites) Form Raft‐like Microdomains and Target Lipid Droplets on the Endoplasmic Reticulum: Roles in Endoplasmic Reticulum Lipid Compartmentalization and Export.” Journal of Pharmacology and Experimental Therapeutics 306, no. 2: 718–725. 10.1124/jpet.103.051284. 12730355

[hup70019-bib-0040] Hayashi, T. , and T. P. Su . 2007. “Sigma‐1 Receptor Chaperones at the ER‐mitochondrion Interface Regulate Ca(2+) Signaling and Cell Survival.” Cell 131, no. 3: 596–610. 10.1016/j.cell.2007.08.036. 17981125

[hup70019-bib-0041] Herrando‐Grabulosa, M. , N. Gaja‐Capdevila , J. M. Vela , and X. Navarro . 2021. “Sigma 1 Receptor as a Therapeutic Target for Amyotrophic Lateral Sclerosis.” British Journal of Pharmacology 178, no. 6: 1336–1352. 10.1111/bph.15224. 32761823

[hup70019-bib-0042] Hershey, T. , H. M. Lugar , J. S. Shimony , et al., and Washington University Wolfram Study Group . 2012. “Early Brain Vulnerability in Wolfram syndrome.” PLoS One 7, no. 7: e40604. 10.1371/journal.pone.0040604. 22792385 PMC3394712

[hup70019-bib-0043] Hotta, K. , T. Funahashi , N. L. Bodkin , et al. 2001. “Circulating Concentrations of the Adipocyte Protein Adiponectin Are Decreased in Parallel With Reduced Insulin Sensitivity During the Progression to Type 2 Diabetes in Rhesus Monkeys.” Diabetes 50, no. 5: 1126–1133. 10.2337/diabetes.50.5.1126. 11334417

[hup70019-bib-0044] Hustveit, O. , A. Maurset , and I. Oye . 1995. “Interaction of the Chiral Forms of Ketamine With Opioid, Phencyclidine, Sigma and Muscarinic Receptors.” Pharmacology & Toxicology 77, no. 6: 355–359. 10.1111/j.1600-0773.1995.tb01041.x. 8835358

[hup70019-bib-0045] Johnston, J. N. , I. D. Henter , and C. A. Zarate Jr . 2023. “The Antidepressant Actions of Ketamine and Its Enantiomers.” Pharmacology & Therapeutics 246: 108431. 10.1016/j.pharmthera.2023.108431. 37146727 PMC10213151

[hup70019-bib-0046] Kakiuchi, C. , S. Ishigaki , C. M. Oslowski , S. G. Fonseca , T. Kato , and F. Urano . 2009. “Valproate, a Mood Stabilizer, Induces WFS1 Expression and Modulates Its Interaction With ER Stress Protein GRP94.” PLoS One 4, no. 1: e4134. 10.1371/journal.pone.0004134. 19125190 PMC2607540

[hup70019-bib-0047] Kang, M. J. Y. , E. Hawken , and G. H. Vazquez . 2022. “The Mechanisms Behind Rapid Antidepressant Effects of Ketamine: A Systematic Review With a Focus on Molecular Neuroplasticity.” Frontiers in Psychiatry 13: 860882. 10.3389/fpsyt.2022.860882. 35546951 PMC9082546

[hup70019-bib-0048] Kinsley, B. T. , M. Swift , R. H. Dumont , and R. G. Swift . 1995. “Morbidity and Mortality in the Wolfram Syndrome.” Diabetes Care 18, no. 12: 1566–1570. 10.2337/diacare.18.12.1566. 8722052

[hup70019-bib-0049] Koh, P. O. , A. S. Undie , N. Kabbani , R. Levenson , P. S. Goldman‐Rakic , and M. S. Lidow . 2003. “Up‐Regulation of Neuronal Calcium Sensor‐1 (NCS‐1) in the Prefrontal Cortex of Schizophrenic and Bipolar Patients.” Proceedings of the National Academy of Sciences of the United States of America 100, no. 1: 313–317. 10.1073/pnas.232693499. 12496348 PMC140961

[hup70019-bib-0050] Koido, K. , S. Kõks , T. Nikopensius , et al. 2005. “Polymorphisms in Wolframin (WFS1) Gene Are Possibly Related to Increased Risk for Mood Disorders.” International Journal of Neuropsychopharmacology 8, no. 2: 235–244. 10.1017/S1461145704004791. 15473915

[hup70019-bib-0051] Kõks, S. 2023. “Genomics of Wolfram Syndrome 1 (WFS1).” Biomolecules 13, no. 9: 1346. 10.3390/biom13091346. 37759745 PMC10527379

[hup70019-bib-0052] Kondo, M. , K. Tanabe , K. Amo‐Shiinoki , et al. 2018. “Activation of GLP‐1 Receptor Signalling Alleviates Cellular Stresses and Improves Beta Cell Function in a Mouse Model of Wolfram Syndrome.” Diabetologia 61, no. 10: 2189–2201. 10.1007/s00125-018-4679-y. 30054673

[hup70019-bib-0053] Koponen, H. , H. Kautiainen , E. Leppänen , P. Mäntyselkä , and M. Vanhala . 2015. “Association Between Suicidal Behaviour and Impaired Glucose Metabolism in Depressive Disorders.” BMC Psychiatry 15, no. 1: 163. 10.1186/s12888-015-0567-x. 26199013 PMC4509469

[hup70019-bib-0054] Kopp, H. P. , K. Krzyzanowska , M. Möhlig , J. Spranger , A. F. Pfeiffer , and G. Schernthaner . 2005. “Effects of Marked Weight Loss on Plasma Levels of Adiponectin, Markers of Chronic Subclinical Inflammation and Insulin Resistance in Morbidly Obese Women.” International Journal of Obesity 29, no. 7: 766–771. 10.1038/sj.ijo.0802983. 15917853

[hup70019-bib-0055] Leal, G. C. , I. D. Bandeira , F. S. Correia‐Melo , et al. 2021. “Intravenous Arketamine for treatment‐resistant Depression: Open‐Label Pilot Study.” European Archives of Psychiatry and Clinical Neuroscience 271, no. 3: 577–582. 10.1007/s00406-020-01110-5. 32078034

[hup70019-bib-0056] Lenart, L. , J. Hodrea , A. Hosszu , et al. 2016. “The Role of Sigma‐1 Receptor and Brain‐Derived Neurotrophic Factor in the Development of Diabetes and Comorbid Depression in Streptozotocin‐Induced Diabetic Rats.” Psychopharmacology 233, no. 7: 1269–1278. 10.1007/s00213-016-4209-x. 26809458

[hup70019-bib-0057] Liiv, M. , A. Vaarmann , D. Safiulina , et al. 2024. “ER Calcium Depletion as a Key Driver for Impaired ER‐to‐Mitochondria Calcium Transfer and Mitochondrial Dysfunction in Wolfram Syndrome.” Nature Communications 15, no. 1: 6143. 10.1038/s41467-024-50502-x. PMC11271478 39034309

[hup70019-bib-0058] Lu, S. , K. Kanekura , T. Hara , et al. 2014. “A Calcium‐Dependent Protease as a Potential Therapeutic Target for Wolfram Syndrome.” Proceedings of the National Academy of Sciences of the United States of America 111, no. 49: E5292–E5301. 10.1073/pnas.1421055111. 25422446 PMC4267371

[hup70019-bib-0059] Ma, H. , J. F. Li , X. Qiao , et al. 2024. “Sigma‐1 Receptor Activation Mediates the Sustained Antidepressant Effect of Ketamine in Mice Via Increasing BDNF Levels.” Acta Pharmacologica Sinica 45, no. 4: 704–713. 10.1038/s41401-023-01201-8. 38097715 PMC10943013

[hup70019-bib-0060] Machado‐Vieira, R. , P. W. Gold , D. A. Luckenbaugh , et al. 2017. “The Role of Adipokines in the Rapid Antidepressant Effects of Ketamine.” Molecular Psychiatry 22, no. 1: 127–133. 10.1038/mp.2016.36. 27046644 PMC5112162

[hup70019-bib-0061] Malar, D. S. , P. Thitilertdecha , K. S. Ruckvongacheep , S. Brimson , T. Tencomnao , and J. M. Brimson . 2023. “Targeting Sigma Receptors for the Treatment of Neurodegenerative and Neurodevelopmental Disorders.” CNS Drugs 37, no. 5: 399–440. 10.1007/s40263-023-01007-6. 37166702 PMC10173947

[hup70019-bib-0062] Marcantoni, W. S. , B. S. Akoumba , M. Wassef , et al. 2020. “A Systematic Review and Meta‐Analysis of the Efficacy of Intravenous Ketamine Infusion for Treatment Resistant Depression: January 2009 ‐ January 2019.” Journal of Affective Disorders 277: 831–841. 10.1016/j.jad.2020.09.007. 33065824

[hup70019-bib-0063] Mirfazeli, F. S. , F. Mohebi , A. Jahanbakhshi , O. Aryani , and M. Almasi‐Dooghaee . 2022. “Repetitive Suicidal Behaviors in a Case With a New Mutation of Wolfram syndrome: A Jump From the Gene to the Behavior.” Basic and Clinical Neuroscience 13, no. 6: 893–900. 10.32598/bcn.2021.910.3. 37323959 PMC10262289

[hup70019-bib-0064] Motawe, Z. Y. , S. S. Abdelmaboud , J. Cuevas , and J. W. Breslin . 2020. “PRE‐084 as a Tool to Uncover Potential Therapeutic Applications for Selective Sigma‐1 Receptor Activation.” International Journal of Biochemistry & Cell Biology 126: 105803. 10.1016/j.biocel.2020.105803. 32668330 PMC7484451

[hup70019-bib-0065] Narita, M. , K. Yoshizawa , K. Aoki , M. Takagi , M. Miyatake , and T. Suzuki . 2001. “A Putative Sigma1 Receptor Antagonist NE‐100 Attenuates the Discriminative Stimulus Effects of Ketamine in Rats.” Addiction Biology 6, no. 4: 373–376. 10.1080/13556210020077091. 11900615

[hup70019-bib-0066] Nguyen, L. , B. P. Lucke‐Wold , S. A. Mookerjee , et al. 2015. “Role of Sigma‐1 Receptors in Neurodegenerative Diseases.” Journal of Pharmacological Sciences 127, no. 1: 17–29. 10.1016/j.jphs.2014.12.005. 25704014

[hup70019-bib-0067] Ossa, F. , J. R. Schnell , and J. L. Ortega‐Roldan . 2017. “A Review of the Human Sigma‐1 Receptor Structure.” Advances in Experimental Medicine and Biology 964: 15–29. 10.1007/978-3-319-50174-1_3. 28315262

[hup70019-bib-0068] Oxombre, B. , C. Lee‐Chang , A. Duhamel , et al. 2015. “High‐Affinity Σ1 Protein Agonist Reduces Clinical and Pathological Signs of Experimental Autoimmune Encephalomyelitis.” British Journal of Pharmacology 172, no. 7: 1769–1782. 10.1111/bph.13037. 25521311 PMC4376455

[hup70019-bib-0069] Patergnani, S. , F. Baldassari , E. De Marchi , A. Karkucinska‐Wieckowska , M. R. Wieckowski , and P. Pinton . 2014. “Methods to Monitor and Compare Mitochondrial and Glycolytic ATP Production.” Methods in Enzymology 542: 313–332. 10.1016/B978-0-12-416618-9.00016-9. 24862273

[hup70019-bib-0070] Pergolizzi, J. , G. Varrassi , M. Coleman , et al. 2023. “The Sigma Enigma: A Narrative Review of Sigma Receptors.” Cureus 15, no. 3: e35756. 10.7759/cureus.35756. 37020478 PMC10069457

[hup70019-bib-0071] Pourtoy‐Brasselet, S. , A. Sciauvaud , M. G. Boza‐Moran , et al. 2021. “Human iPSC‐Derived Neurons Reveal Early Developmental Alteration of Neurite Outgrowth in the Late‐Occurring Neurodegenerative Wolfram Syndrome.” American Journal of Human Genetics 108, no. 11: 2171–2185. 10.1016/j.ajhg.2021.10.001. 34699745 PMC8595949

[hup70019-bib-0072] Price, R. B. , D. V. Iosifescu , J. W. Murrough , et al. 2014. “Effects of Ketamine on Explicit and Implicit Suicidal Cognition: A Randomized Controlled Trial in treatment‐resistant Depression.” Depression and Anxiety 31, no. 4: 335–343. 10.1002/da.22253. 24668760 PMC4112410

[hup70019-bib-0073] Ren, P. , J. Wang , N. Li , et al. 2022. “Sigma‐1 Receptors in Depression: Mechanism and Therapeutic Development.” Frontiers in Pharmacology 13: 925879. 10.3389/fphar.2022.925879. 35784746 PMC9243434

[hup70019-bib-0074] Rigoli, L. , P. Bramanti , C. Di Bella , and F. De Luca . 2018. “Genetic and Clinical Aspects of Wolfram syndrome 1, a Severe Neurodegenerative Disease.” Pediatric Research 83, no. 5: 921–929. 10.1038/pr.2018.17. 29774890

[hup70019-bib-0075] Robson, M. J. , M. Elliott , M. J. Seminerio , and R. R. Matsumoto . 2012. “Evaluation of Sigma (σ) Receptors in the Antidepressant‐Like Effects of Ketamine in Vitro and in Vivo.” European Neuropsychopharmacology 22, no. 4: 308–317. 10.1016/j.euroneuro.2011.08.002. 21911285

[hup70019-bib-0076] Salaciak, K. , and K. Pytka . 2022. “Revisiting the Sigma‐1 Receptor as a Biological Target to Treat Affective and Cognitive Disorders.” Neuroscience & Biobehavioral Reviews 132: 1114–1136. 10.1016/j.neubiorev.2021.10.037. 34736882 PMC8559442

[hup70019-bib-0077] Sánchez, J. C. , A. Alemán , J. F. Henao , J. C. Olaya , and B. E. Ehrlich . 2024. “NCS‐1 Protein Regulates TRPA1 Channel Through the PI3K Pathway in Breast Cancer and Neuronal Cells.” Journal of Physiology & Biochemistry 80, no. 2: 451–463. 10.1007/s13105-024-01016-z. 38564162 PMC11074019

[hup70019-bib-0078] Schmidt, H. R. , and A. C. Kruse . 2019. “The Molecular Function of σ Receptors: Past, Present, and Future.” Trends in Pharmacological Sciences 40, no. 9: 636–654. 10.1016/j.tips.2019.07.006. 31387763 PMC6748033

[hup70019-bib-0079] Scotton, E. , B. Antqueviezc , M. F. Vasconcelos , et al. 2022. “Is (R)‐Ketamine a Potential Therapeutic Agent for Treatment‐Resistant Depression With Less Detrimental Side Effects? A Review of Molecular Mechanisms Underlying Ketamine and Its Enantiomers.” Biochemical Pharmacology 198: 114963. 10.1016/j.bcp.2022.114963. 35182519

[hup70019-bib-0080] Seppa, K. , M. Toots , R. Reimets , et al. 2019. “GLP‐1 Receptor Agonist Liraglutide Has a Neuroprotective Effect on an Aged Rat Model of Wolfram Syndrome.” Scientific Reports 9, no. 1: 15742. 10.1038/s41598-019-52295-2. 31673100 PMC6823542

[hup70019-bib-0081] Sequeira, A. , C. Kim , M. Seguin , et al. 2003. “Wolfram syndrome and Suicide: Evidence for a Role of WFS1 in Suicidal and Impulsive Behavior.” American Journal of Medical Genetics Part B, Neuropsychiatric genetics: the official publication of the International Society of Psychiatric Genetics 119B, no. 1: 108–113. 10.1002/ajmg.b.20011. 12707947

[hup70019-bib-0082] Singh, B. , S. Kung , V. Pazdernik , et al. 2023. “Comparative Effectiveness of Intravenous Ketamine and Intranasal Esketamine in Clinical Practice Among Patients With Treatment‐Refractory Depression: An Observational Study.” Journal of Clinical Psychiatry 84, no. 2: 22m14548. 10.4088/JCP.22m14548. 36724113

[hup70019-bib-0083] Swift, M. , and R. G. Swift . 2005. “Wolframin Mutations and Hospitalization for Psychiatric Illness.” Molecular Psychiatry 10, no. 8: 799–803. 10.1038/sj.mp.4001681. 15852062

[hup70019-bib-0084] Swift, R. G. , M. H. Polymeropoulos , R. Torres , and M. Swift . 1998. “Predisposition of Wolfram syndrome Heterozygotes to Psychiatric Illness.” Molecular Psychiatry 3, no. 1: 86–91. 10.1038/sj.mp.4000344. 9491819

[hup70019-bib-0085] Swift, R. G. , D. B. Sadler , and M. Swift . 1990. “Psychiatric Findings in Wolfram Syndrome Homozygotes.” Lancet (London, England) 336, no. 8716: 667–669. 10.1016/0140-6736(90)92157-d. 1975860

[hup70019-bib-0086] Szabo, A. , A. Kovacs , J. Riba , S. Djurovic , E. Rajnavolgyi , and E. Frecska . 2016. “The Endogenous Hallucinogen and Trace Amine N,N‐Dimethyltryptamine (DMT) Displays Potent Protective Effects Against Hypoxia Via Sigma‐1 Receptor Activation in Human Primary iPSC‐Derived Cortical Neurons and Microglia‐like Immune Cells.” Frontiers in Neuroscience 10: 423. 10.3389/fnins.2016.00423. 27683542 PMC5021697

[hup70019-bib-0087] Takahashi, N. , A. Yamada , A. Shiraishi , H. Shimizu , R. Goto , and Y. Tominaga . 2021. “Efficacy and Safety of Fixed Doses of Intranasal Esketamine as an Add‐On Therapy to Oral Antidepressants in Japanese Patients With treatment‐resistant Depression: A Phase 2b Randomized Clinical Study.” BMC Psychiatry 21, no. 1: 526. 10.1186/s12888-021-03538-y. 34696742 PMC8547110

[hup70019-bib-0088] Torres, K. C. , B. R. Souza , D. M. Miranda , et al. 2009. “Expression of Neuronal Calcium Sensor‐1 (NCS‐1) is Decreased in Leukocytes of Schizophrenia and Bipolar Disorder Patients.” Progress in Neuro‐Psychopharmacology and Biological Psychiatry 33, no. 2: 229–234. 10.1016/j.pnpbp.2008.11.011. 19091302

[hup70019-bib-0089] Vollenweider, F. X. , K. L. Leenders , I. Oye , D. Hell , and J. Angst . 1997. “Differential Psychopathology and Patterns of Cerebral Glucose Utilisation Produced by (S)‐ and (R)‐Ketamine in Healthy Volunteers Using Positron Emission Tomography (PET).” European Neuropsychopharmacology 7, no. 1: 25–38. 10.1016/s0924-977x(96)00042-9. 9088882

[hup70019-bib-0090] Voronin, M. V. , Y. V. Vakhitova , and S. B. Seredenin . 2020. “Chaperone Sigma1R and Antidepressant Effect.” International Journal of Molecular Sciences 21, no. 19: 7088. 10.3390/ijms21197088. 32992988 PMC7582751

[hup70019-bib-0091] Wang, Y. , R. W. Meng , S. K. Kunutsor , et al. 2018. “Plasma Adiponectin Levels and Type 2 Diabetes Risk: A Nested Case‐Control Study in a Chinese Population and an Updated Meta‐Analysis.” Scientific Reports 8, no. 1: 406. 10.1038/s41598-017-18709-9. 29321603 PMC5762808

[hup70019-bib-0092] Wang, Y. M. , C. Y. Xia , H. M. Jia , et al. 2022. “Sigma‐1 Receptor: A Potential Target for the Development of Antidepressants.” Neurochemistry International 159: 105390. 10.1016/j.neuint.2022.105390. 35810915

[hup70019-bib-0093] Weng, T. Y. , S. A. Tsai , and T. P. Su . 2017. “Roles of Sigma‐1 Receptors on Mitochondrial Functions Relevant to Neurodegenerative Diseases.” Journal of Biomedical Science 24, no. 1: 74. 10.1186/s12929-017-0380-6. 28917260 PMC5603014

[hup70019-bib-0094] Westling, S. , B. Ahrén , L. Träskman‐Bendz , and A. Westrin . 2004. “High CSF‐Insulin in Violent Suicide Attempters.” Psychiatry Research 129, no. 3: 249–255. 10.1016/j.psychres.2004.09.004. 15661318

[hup70019-bib-0095] Wilkinson, S. T. , E. D. Ballard , M. H. Bloch , et al. 2018. “The Effect of a Single Dose of Intravenous Ketamine on Suicidal Ideation: A Systematic Review and Individual Participant Data Meta‐Analysis.” American Journal of Psychiatry 175, no. 2: 150–158. 10.1176/appi.ajp.2017.17040472. 28969441 PMC5794524

[hup70019-bib-0096] Włodarczyk, A. , J. Słupski , J. Szarmach , and W. J. Cubała . 2024. “Single Arketamine in Treatment Resistant Depression: Presentation of 3 Cases With Regard to Sick‐Leave Duration.” Asian Journal of Psychiatry 96: 104016. 10.1016/j.ajp.2024.104016. 38554563

[hup70019-bib-0097] Yang, C. , Y. Shirayama , J. C. Zhang , et al. 2015. “R‐Ketamine: A Rapid‐Onset and Sustained Antidepressant Without Psychotomimetic Side Effects.” Translational Psychiatry 5, no. 9: e632. 10.1038/tp.2015.136. 26327690 PMC5068814

[hup70019-bib-0098] Yin, J. , M. Li , L. Xu , et al. 2013. “Insulin Resistance Determined by Homeostasis Model Assessment (HOMA) and Associations With Metabolic Syndrome Among Chinese Children and Teenagers.” Diabetology & Metabolic Syndrome 5, no. 1: 71. 10.1186/1758-5996-5-71. 24228769 PMC3833654

[hup70019-bib-0099] Zatyka, M. , T. R. Rosenstock , C. Sun , et al. 2023. “Depletion of WFS1 Compromises Mitochondrial Function in hiPSC‐Derived Neuronal Models of Wolfram syndrome.” Stem Cell Reports 18, no. 5: 1090–1106. 10.1016/j.stemcr.2023.04.002. 37163979 PMC10202695

[hup70019-bib-0100] Zhang, J. , L. Ma , X. Wan , J. Shan , Y. Qu , and K. Hashimoto . 2021. “(R)‐Ketamine Attenuates LPS‐Induced Endotoxin‐Derived Delirium Through Inhibition of Neuroinflammation.” Psychopharmacology 238, no. 10: 2743–2753. 10.1007/s00213-021-05889-6. 34313805

[hup70019-bib-0101] Zhang, J. C. , S. X. Li , and K. Hashimoto . 2014. “R (‐)‐Ketamine Shows Greater Potency and Longer Lasting Antidepressant Effects Than S (+)‐Ketamine.” Pharmacology Biochemistry and Behavior 116: 137–141. 10.1016/j.pbb.2013.11.033. 24316345

